# Optimisation of methods for Agrobacterium rhizogenes mediated generation of composite plants in *Eucalyptus camaldulensis*

**DOI:** 10.1186/1753-6561-5-S7-O45

**Published:** 2011-09-13

**Authors:** Aiyar Balasubramanian, Rathinavel Venkatachalam, K R Selvakesavan, Abraham Sanu Mary, Hassen Gherbi, Sergio Svistoonoff, Claudine Franche, Didier Bogusz, N Krishna Kumar, Mathish Nambiar-Veetil

**Affiliations:** 1 Institute of Forest Genetics and Tree Breeding, Coimbatore, Tamil Nadu-641002, India; 2 Institut de Recherche pour le Développement (IRD), Montpellier-34394, France

## Background

The wide adaptability, fast growth rate, good form, excellent wood and fiber properties make *Eucalyptus* the most widely planted genus of plantation forest trees. With the availability of Eucalyptus genome sequence information, assignment of function to genes contributing to desired traits require functional validation via overexpression or silencing in the context of the plant environment. However, plant regeneration from transformed tissues has been the most time consuming and rate-limiting step for rapid gene function analysis in tree species. The composite plants with transgenic hairy roots derived via *Agrobacterium rhizogenes* mediated transformation are therefore increasingly being used in species with such limitations to study root development and root-biotic interactions [1]. In this paper we describe a protocol developed for generation of composite plants using *A. rhizogenes* mediated transformation of *Eucalyptus camaldulensis*.

## Methods

Transformation experiments were carried out using an agropine mannopine-type strain A4RS, harbouring a green fluorescent protein (sGFP) based binary vector pHKN29, constructed from pCAMBIA 1300 by replacing hygromycin phosphotransferase (hpt) with the sGFP(S65T) [2]. Infection was carried out using a sterile needle swabbed with *A. rhizogenes* grown for 48 hours in YEB plates containing 100 µM Acetosyringone. The infected plants of *E. camaldulensis* were then placed under dim light on modified MS [3] medium comprising half strength MS macro elements, full strength MS minor elements and organic supplements. The petridishes were placed slantingly with the plants in an upside-down position. Following cocultivation, the cultures were transferred to fresh medium with plants in an upright position. Hairy roots were made bacteria-free by making transfers every 15 days to fresh medium containing 500mg/l cefotaxime. The hairy roots generated were examined under a stereo fluorescence microscope (Nikon, Japan) for visualization of GFP following excitation by blue light (Excitation filter 495nm, Barrier filter 520nm). The nontransformed roots were removed using sterile blades. The composite plants were placed in test tubes, and different media including full MS, modified MS, Knop’s, B5 and Hoagland’s solutions were evaluated for growth under hydroponic conditions. The composite plants were shifted to the transgenic greenhouse, where in the cotton plugs were loosened gradually and finally removed after a period of 2 weeks. The plants were then placed so that roots were immersed in the liquid media and shoots were above the test tube rim covered with aluminium foil. Few of these plants were subjected to stepwise increasing concentration of 50mM NaCl to determine their tolerance to salt stress.

## Results and discussion

**Figure 1:**
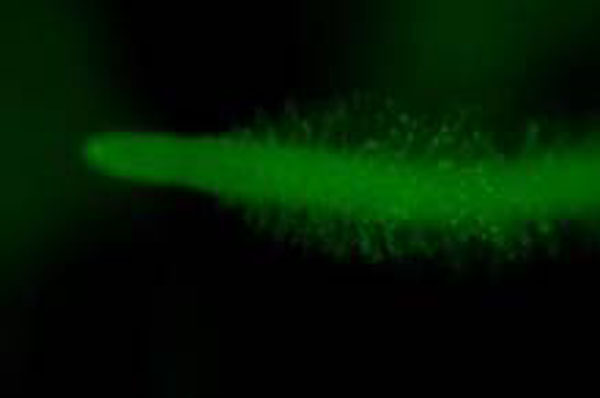
GFP expressing hairy root of *E. camaldulensis*

Inoculation with A4RS resulted in roots, arising mainly from the injured surface of the explants. Root initiation occurred within first 7 days after inoculation of explants with *A. rhizogenes.* The roots reached a length of 8-10cm in 45 days. The results were similar to the report of MacRae and Van Staden (1993), where in genetic transformation using the *A. rhizogenes* strain LBA 9402 was assessed by the detection of opines in the root generated from *in vitro* propagated seedling explants of *E. grandis*, *E. dunni* and *E. nitens*. In our study, the bright green fluorescence due to co-transformation of the pHKN29 vector harbouring the sGFP(S65T) was used for screening of transgenic roots (Figure 1). No fluorescence was observed in untransformed control roots of Eucalyptus.

## Effect of explant type

Seedlings showed root induction in 68 % of the explants at an average of 1.08 roots per plant of which 36 % were cotransformed with the pHKN29 as confirmed by the GFP expression. The *in vitro* propagated clonal plantlets showed root induction in 37.5 % of the explants at an average of 0.45 roots per plant of which 4.1% showed GFP expression.

## Effect of cocultivation conditions

**Figure 2:**
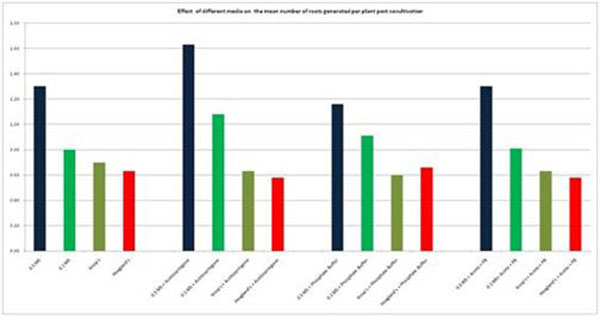
Effect of different media / supplements on the average number of roots generated per plant post cocultivation

In a separate experiment, out of the four different basal media used for cocultivation, modified MS media gave the highest average number of hairy roots at 1.3 per plant, when compared to 0.8, 0.7 and 0.6 in case of 0.1 MS, Knop’s and Hoagland’s media (Figure 2).

Different supplements like phosphate buffer and calcium chloride were included in the cocultivation media to evaluate their influence on hairy root induction. Addition of calcium chloride to the cocultivation media reduced the average number of hairy roots induced per plant by 30%. Similarly, phosphate buffer also did not significantly influence the hairy root generation.

However, the inclusion of 100 µM acetosyringone in the cocultivation medium increased the average number of hairy roots to 1.6 per plant as compared to 1.3 for media without acetosyringone (Figure 2). Acetosyringone has been known to enhance transformation efficiency through activation of *vir* genes in *Agrobacterium*.

Preliminary experiments indicated that, cotransformation efficiency was higher when cocultivation was carried out at 16.5°C for 14 days when compared to 22°C for 7 days.

## Effect of different liquid media on hardening of composite plants

Different liquid media were evaluated for hardening of composite plantlets. Hoagland’s solution showed the highest survival (73.3%) when compared to MS (20%), modified MS (16%), B5 (0%) and Knop’s (3.3%). To determine the tolerance of these composite plants under salt stress, they were subjected to weekly increase in the concentration of NaCl by 50mM. Fifty percent of the Eucalyptus composite plants were able to tolerate up to 300 mM NaCl stress.

## Conclusion

A composite plant strategy was developed utilising GFP based screening of transgenic hairy roots. Different parameters were evaluated to increase transformation efficiency using *A. rhizogenes*. Our studies showed that, modified MS media containing 100 µm Acetosyringone was optimum for enhancing the cotransformation efficiency. Hoagland’s solution was identified as an appropriate media for hardening of Eucalyptus composite plants under transgenic green house conditions. Roots being the major portal of entry of ions to plants, the composite plant strategy will provide a rapid tool for the functional analysis of the genes vital for ion transport and root development. The composite plant strategy will be used to validate the function of *EcHKT1/EtHKT1*, the homologue of which have been reported to contribute to Na^+^ uptake and xylem unloading of Na^+^ in model plant and crop species.
